# The American Medical Association’s Next Meeting

**Published:** 1856-03

**Authors:** 


### ART. III.—*The American Medical Association's Next Meeting. What is to be done.*

But a comparatively short time intervenes between this and the date of the Annual Meeting of the American Medical Association, which convenes on the first Tuesday of May next, in the city of Detroit. As this organization may fairly assume to be the great head of the profession in our country, and in its acts speak forth the embodied sentiments of the profession in ethics and practice, its prospective action is not unworthy the attention of every individual practitioner whose interests by its legislative enactments, or by its adoption and promulgation of theories may be affected thereby, in order that hasty or injudicious action may not lead to the injury of the profession, instead of its advancement. Our near proximity to the place of its next assemblage will undoubtedly invest the convocation with greater interest to those in this section, and it is not unreasonable to suppose that the western sections of our union will be largely represented among the several delegations, and that very many, for the first time, will assume a part in the deliberations of this body. From the nature of the organization of the association, and the migratory manner of ranging over the length and breadth of our widely extended national domains, in its places of meeting, each assemblage must necessarily embrace many *new* men, called upon at the moment to decide questions of the deepest interest and importance to the profession. While this ever changing character in the *materiel* of the association, undoubtedly imparts greater vigor to its constitution, and, perhaps, affords a guarantee against decay, while it does most assuredly secure a real, living expression of the sentiments of the great and widely dispersed mass of the profession, and guards against its becoming the mere mouthpiece of sectional medicine, or the exponent of localized fogyism; still it is from this very cause not without its dangers of sometimes acting hastily and without sufficient consideration. But little time can be given for deliberation in the multiplicity of objects claiming the attention of each session. Although "*previous notice*" may be given of intended action, but too few will remember after a cursory reading of the reported proceedings, what subjects are to claim their attention, and the *ax* may be ground, if there be *a grinder* present.

At the risk of the charge of being over-observing, or studious above our neighbors, we propose making a note of the various matters submitted at the last meeting of the association to be discussed at the coming session. The more appropriate will this appear, when the fact is taken into consideration, that all the subjects calculated to affect the present mode of organization of the association, and its established customs, such as constitutional amendments and the like, were deferred over to, and pressed into the proceedings of the last day of the session, when a smaller number was in attendance than on any previous day. The truth of this any one may verify for himself by an examination of the proceedings of the meeting published in the volume of Transactions.

A proposition was on that day submitted by Dr. N. S. Davis, consisting of several preambles and resolutions; one part of which for the carrying out of the designs of the organization in its scientific capacity, proposes the division of each day of the session into two parts, one of which is to be exclusively devoted to the general business of the association; the other part to be devoted to scientific purposes alone. To this part of the proposition there is no objection, and its adoption would no doubt add to the interests of the session, and greatly enhance its value. But to the following resolution, forming part of the series in the proposition, we do not so readily accord our acquiescence in its merits. The resolution reads as follows—we have, however, taken the liberty to emphasize it by *italics:*

"*Resolved,* That the association, *in its capacity of a scientific body*, having no power to act on any subject except of a scientific character, *may continue in session*, whenever thought advisable, *a longer period* than in its more *general capacity*."

However plausible, upon first sight, this resolutiou may appear, in its pretext of aiding the cause of scientific discussion, and in the development of truth and the general advancement of medicine, its true result will be to enable those who have but little to demand their attention at home, to prolong the session needlessly, and to remain at last after it has adjourned "in its more general capacity," until they have themselves finally become tired of pluming themselves before the public in the name of the American Medical Association. We confess we have a great dislike to the proposed innovation upon the old custom. A vision rises before us of the daily convocations thus spun out, where the few who linger are resplendent in all the glories of the bright name under which they cluster; and relieved from the impertinent observations and remarks of the crowd who have gone *about their bus-*

*iness*, they complacently settle themselves down into a mutual admiration society, and their lucubrations rival only in luminosity the discussions of the far-famed Farmer's Clubs of Gotham.

Let, we pray, whatever is done by the association, be done "in its more general capacity." If a session of four days be not enough to accomplish what is desired, let it be so known, and prolong the session into the second week. The members with a foreknowledge of such a necessity, would adapt themselves to the circumstances of the case, and would also carefully guard against any loss of time by devotions at the shrine of *buncomb*.

The following resolution was offered by Dr. Foster, and laid upon the table:

"*Resolved*, That the State Medical Societies be requested to hold their annual meetings just one month before the meeting of the American Medical Association."

It will be a pity to disturb the repose of this resolution. It is difficult to conceive of any possible good to come of it, and its execution is perfectly impracticable.

A proposition to be reported on, having for its object an inquiry into an alteration of the manner of imparting instruction by lectures, was submitted. It proposes the extension of the same course of lectures over two terms. As this subject more immediately interests our Medical Colleges, and they will doubtless keep their eyes open to whatever may affect their interests, we will leave the subject to their care.

The following should arrest the attention of every practitioner in the State of New York, at least. In order to display the subject in its full length and breadth, we make the following extract at length from page fifty-eight of the volume of Transactions for 1855:

"Dr. Levi D. Sheets, of Ohio, offered the following preamble and resolutions, which, as an amendment of the constitution, lie over, under the rule, until the next annual meeting of the association:

"*Whereas*, it has been found necessary to devise some means of elevating the standard of education, both professional and general, among medical men, by those who are best acquainted with the sad and lamentable deficiency which prevails in this respect among a large mass of the profession, and particularly in the we[illegible]rn states.

"*And whereas*, efforts to this effect, at home, have been opposed on the ground that more was exacted by such as took an active part in the matter than is required by the 'American Medical Association.'

"*And whereas*, it is believed that the National Society can exert a beneficial influence over the whole country; therefore

"*Resolved*, That the constitution of the association be so amended as to require all delegates before being allowed a seat in said association, to satisfy the proper authorities that the societies which they represent, require *graduation* as the *sine quâ non* to membership therein, and that no person can become a *permanent member*, a *member by invitation*, or can be received as a *delegate* from any other body, *unless he be a graduate* of some respectable medical school.

"*Resolved*, That the editors of the various medical journals of the United States, be requested to publish the foregoing, so that an interest may be awakened upon this subject, and that societies may be prepared to comply with the above requisitions in case they meet the approval of this association."

We repeat that the above proposed amendment to the constitution should arrest the attention of every regular practitioner in this state. For the effect of it is, if adopted, to disfranchise the entire faculty of the state, and render them ineligible to membership with the association. Whatever might be its effects elsewhere, such would be the result here. Our county and state societies *do not* require *graduation* as a *sine quâ non* to membership, nor will they. Under the statutes of the states which give them their existence, they have the power to graduate, or as they more modestly express it, to *grant licenses to practice medicine and surgery*. These licenses confer in this state all the rights and privileges which the medical schools can grant. These societies have been in the exercise of this power for near half a century, and it is too late now to ask them to dispossess themselves of rights and privileges granted them so long since, and which in all their multitudinous provisions have been time and again confirmed to them by our courts. And the more particularly, in the absence of any good reason for the surrender of this prerogative, is such a concession not to be expected. The proposer of this reform in the deficiencies in the qualifications of a "large mass of the profession," has commenced his labors in too indiscriminate and reckless a manner to accomplish much good. We do not care to suffer in this state for the shortcomings of others. Whatever may be the condition of things in the "western states particularly" as is referred to in the resolution, there exists here no legal difference, or value between the diploma of the colleges and the license of the county society. The possession of the one instead of the other, is less a measure of attainments than of the pecuniary abilities of the student. And as many of the medical reformers of the day do not hesitate to charge that the possession of a diploma does not necessarily prove the qualification of its recipient and holder, neither, we affirm, does the possession of a license *per se*, prove its holder a blockhead.

Nothing but the grossest carelessness can permit so unjust and injurious an alteration in the constitution of the association. We think we could hardly have had a more striking example to illustrate the proposition with which we introduced this article—the danger to be apprehended from hasty and injudicious action.

Another amendment of the constitution also lies over, proposing "that the part of the constitution which relates to the election of officers be so amended that the election shall take place immediately before its adjournment, instead of immediately after its commencement," in order that the presiding officer may, during the long recess between the meetings, be able to acquaint himself more perfectly with parliamentary usages and laws.

A proposition to change the day of meeting from the first to the second Tuesday in May, lies also on the table.

An addition to the second article of the constitution was proposed by Dr. W. B. Ives, giving power "to punish any of its members by reprimand, suspension, or expulsion, upon a three-fourths vote of the members present at any meeting, at which not less than one hundred are in attendance."

"Dr. Dorset, of Virginia, offered the following amendment to the constitution:

"*Resolved*, That with a view of adding dignity and influence to our body, and conveying a more adequate idea of its national representative character, the title "National Medical Congress" be substituted for that of "American Medical Association," and that we hold our sessions at least once in three years, in Washington, D. C."

We beg leave to suggest that it is a debateable question whether it would add anything to the "dignity and influence of our body," in these days of political degeneracy, to dub it the *National Medical Congress*. And we doubt farther whether this latter term is any more expressive of its nationality than our old name, *American*. Its name now conveys to the ear the full import of its broad, national character, and the new name would do no more, and besides the history and literature of the association is written in its old title, and any change would lead only to confusion.

What benefits are to be gained by a triennial pilgrimage to Washington, we are entirely ignorant of. The subject of permanently locating somewhere was broached at the last session, and Washington, among other places, was spoken of. The effects of permanently locating deserve careful consideration, as such a change would make a strong impression, either for good, or evil.

A multitude of committees were appointed to report upon strictly medical subjects, of which if a quarter only report, will furnish ample employment for the time of the association.

A committee was also appointed to endeavor to cheapen the cost of traveling to the delegates attending the session. It is much to be hoped that success will attend their labors. Dr. James P. White, represents New York upon the committee.

Might not some of those liberal editors who are so positive of the duty of the physician to work for the public for nothing, under penalty of being assailed in their columns, lend the use of their powerful and *liberal* pens to enforce a reciprocation of those fiee labors by a tender of free pass?

